# Editorial: Adaptations to Advanced Resistance Training Strategies in Youth and Adult Athletes

**DOI:** 10.3389/fphys.2022.888118

**Published:** 2022-03-31

**Authors:** Olaf Prieske, Helmi Chaabene, Jason Moran, Atle H. Saeterbakken

**Affiliations:** ^1^ Division of Exercise and Movement, University of Applied Sciences for Sports and Management Potsdam, Potsdam, Germany; ^2^ Department of Sports and Health Sciences, Faculty of Human Sciences, University of Potsdam, Potsdam, Germany; ^3^ School of Sport, Rehabilitation and Exercise Sciences, University of Essex, Colchester, Essex, United Kingdom; ^4^ Department of Sport, Food and Natural Sciences, Western Norway University of Applied Sciences, Sogndal, Norway

**Keywords:** strength training, sportsmen, mechanisms, chronic effects, acute effects, applied physiology

“Resistance training” (RT), also termed “strength” or “weight training”, has become one of the most popular types of exercise in recent times (Fleck and Kraemer, 2014). Specifically, RT refers to a specialized method of physical conditioning that involves the progressive use of a wide range of resistive loads, including body mass, and a variety of modalities such as machine-based training, free weight training, or plyometric training, to enhance physical fitness, sports-specific performance, and health (Faigenbaum and Myer, 2010; Fleck and Kraemer, 2014). There is abundant evidence on the effectiveness of RT programs on components of physical fitness (e.g., muscle strength, linear speed, change-of-direction speed), sports-specific performance (e.g., throwing/kicking velocity), and health (e.g., injury prevention) in young, as well as adult athletes (Faigenbaum et al., 2016; Lesinski et al., 2016; Moran et al., 2016; Lauersen et al., 2018; Chaabene et al., 2020; Saeterbakken et al., 2022). Accordingly, RT has been recommended as an important training type that should be integrated into all the stages of long-term athlete development to underpin optimal preparation in team and individual sports alike (Lloyd and Oliver, 2012; Granacher et al., 2016).

Of note, Rhea et al. (2003) demonstrated that training status is an important moderator variable in relation to RT-inducing adaptations with an apparent inverse relationship between training status and RT-related gains. In such cases, more advanced RT programs are necessary to provide sufficient training stimuli to maximise the chances of continued adaptation to this form of training (Kraemer and Ratamess, 2004; Schoenfeld et al., 2021). Advanced RT may constitute non-conventional RT methods and overload techniques such as superset training, whole-body/local vibration training, neuromuscular electrical stimulation training, complex training, and blood-flow restriction training (Krzysztofik et al., 2019; Schoenfeld et al., 2021). However, the effectiveness of these methods in improving physical fitness and sports-specific performance, as well as their underpinning mechanisms, are yet not fully described in youth and adult athletes. Therefore, this Research Topic in *Frontiers in Physiology* entitled “*Adaptations to Advanced Resistance Training Strategies in Youth and Adult Athletes*” aimed to gather knowledge on the effects (acute responses and/or chronic adaptations) of advanced RT on components of physical fitness, sports-specific performance and/or health, and their respective underlying mechanisms, on youth and adult athletes.

At the conclusion of this work, a total of fifty-one international authors from Africa, Asia, Australia, Europe, and South America, researching advanced RT strategies, contributed nine peer-reviewed articles to the Research Topic. In terms of article type, six original articles (cross-sectional, longitudinal), one systematic review with meta-analysis, one opinion article and one perspective article were included. A summary of the published works is displayed in Table 1.

**TABLE 1:** Summary of all studies within the Research Topic including type of article, study design, athletes included, research objectives, and main findings.

References	Type of article	Study design	Athletes included	Research objective(s)	Main finding(s)
Aguilera-Castells et al.	Original research	Cross-sectional	Physically active individuals	To examine the effects of vibration during dynamic suspended exercise on muscle activity and perceived exertion	25 Hz vibration during the suspended supine bridge induced higher muscle activity and perceived exertion
Aloui et al.	Original research	RCT	Adolescent soccer players	To examine the effects of 8-week combined plyometric and short sprint training in youth soccer on physical fitness	Combined plyometric and short sprint training improved jump, linear sprint, change-of-direction, repeated sprint, and balance performances
Gentil et al.	Opinion	NA	NA	To discuss benefits and limitations of high-intensity multimodal training programs (e.g., CrossFit) in youth	When professionally supervised, high-intensity multimodal training can be an effective and safe means to improve fitness in youth.
Hamarsland et al.	Original research	RCT	Resistance-trained individuals	To compare the effects of volume-equated, 9-week resistance training frequency (2 vs. 4 x/wk) on gains in muscle strength and mass	Resistance training enhanced muscle strength and mass, irrespective from training frequency.
Mueller et al.	Original research	RCT	Adolescent athletes	To examine the effects of a 6-week trunk-specific sensorimotor vs. resistance training on trunk muscle strength and stability	Both training programs did not induce significant pre-post test changes in trunk muscle strength and stability.
Ramachandran et al.	Systematic review	Systematic literature review with meta-analysis	Healthy individuals	To systematically review and aggregate the effects of plyometric training on measures of balance	Plyometric training enhances static and dynamic balance, irrespective of participants’ sex and age.
Sato et al.	Original research	RCT	Healthy university students	To compare the effects of 5 weeks of unilateral arm curl resistance training at different joint angles on elbow flexors strength and muscle thickness of the trained and non-trained arms	Unilateral arm curl resistance training at extended elbow joints induces greater muscle strength and thickness gains in the trained and untraining arm at extended elbow joint.
Schoeb et al.	Original research	Controlled trial	Youth alpine skiers	To introduce and evaluate the effects of a novel, 12-month injury prevention program on injury incidence	The injury prevention program reduced absolute injury rate and injury incidence rate.
Williams et al.	Perspective	Narrative review	NA	To explore the potential for parkour-based activities in the long-term athlete development of youth basketball players	Parkour could augment youth basketball players’ movement skills and facilitate the transfer of conventional strength and conditioning forms to sport-specific skills

NA = not applicable; RCT = randomized controlled trial

In a cross-sectional study, Aguilera-Castells et al. examined the effects of vibrations, superimposed on to dynamic lower limb suspension exercises, on leg muscle activity in trained individuals. Men and women with approximately 4 years of suspension training experience performed suspended supine bridge and hamstring curl exercises with the legs attached to a suspension system. Vibrations at 25 and 40 Hz were applied during the suspension exercises whilst a ‘no vibration’ condition was also used. Higher muscle activity (i.e., gastrocnemius, semitendinosus) was observed during the suspended supine bridge exercise with superimposed vibrations. This was particularly apparent at 25 Hz when compared to the “no vibration” condition. It was concluded that the suspended supine bridge with superimposed vibration induced a higher stability requirement thus increasing the stabilizing role of the gastrocnemius and semitendinosus muscles.

In a randomized controlled trial, Hamarsland et al. studied the effects of RT frequency on measures of muscle strength and body composition in resistance-trained individuals. Participants conducted 9 weeks of progressive whole-body RT with a frequency of either two or four sessions per week but equal volume. Both training groups improved muscle strength and lean body mass to the same extent, irrespective of training frequency. Additionally, strength gains were more pronounced in less complex exercises than they were in more complex ones (i.e., hack squat over squat, chest press over bench press).

Williams et al. conducted a narrative review with the purpose of exploring the potential for parkour-based activities to be used as part of the long term athletic development of youth basketball players. It was argued that conventional training programs may insufficiently develop fundamental movement skills and the associated transfer to sports-specific tasks due to a narrow range of foundational movement and a lack of decision-making properties. Parkour was characterized by diverse and creative movements used to navigate through an exercise or an obstacle course. With reference to an ecological dynamics perspective, this may facilitate the development of fundamental movement skills and the transfer (i.e., “donation”) of skills and abilities to other sports such as basketball. Complex training was suggested as a feasible training modality to be performed using parkour actions within the same training session as conventional RT exercises.

From a health-related perspective, Schoeb et al. investigated the effects of a novel injury prevention program in alpine skiing on the rate and incidence of injuries in young skiers. For a 12-month intervention period, young competitive alpine skiers in the intervention group performed an injury prevention program, specifically designed for the injury patterns observed in youth skiing (called INSPA_Int_), in addition to their regular training. Skiers in the control group followed their regular training only. The INSPA_Int_ program was designed as a 20 min home-based training session (with online/offline support) and focused on the strengthening of hamstring muscles (eccentric muscle actions), external hip rotators, and trunk muscles. The absolute rates of traumatic and overuse injuries were reduced by 33.5 and 30.1% respectively in the intervention compared with the control group. Moreover, the incidence rate of overuse injury was lowered by 40.2% in the intervention group.

The nine articles in this Research Topic facilitated insight into the large field of RT and advanced strategies with the overarching goal being to detail sufficient training stimuli and to ensure ways to underpin further adaptation(s) in trained individuals. The scope of the advanced RT strategies ranged from variations in training determinants (i.e., training frequency), the inclusion of additional training tools (i.e., vibratory system) to conceptual frameworks in RT (i.e., Parkour, CrossFit). However, it must be highlighted that the conceptual frameworks are currently theory-driven and must therefore be validated as advanced RT strategies in future investigations. Moreover, only Aguilera-Castells et al. and Hamarsland et al. examined mechanistic measures of muscle activity and body composition, respectively, as study outcomes and this is an area that requires further attention in future original studies. Of note, RT-induced performance gains are frequently attributed to changes in muscle activity and/or muscle mass (Behm, 1995; Suchomel et al., 2018). Therefore, future research is still needed to understand the composition and subsequent effects of advanced RT programs, with particular emphasis on longitudinal studies which address both performance and mechanistic outcome measures.
